# Feasibility of automated AI-based contouring and stable radiomic feature assessment by HyperSight-CBCT Imaging for adaptive high-precision radiotherapy of prostate cancer

**DOI:** 10.1038/s41598-026-46359-3

**Published:** 2026-04-11

**Authors:** Ralf Schmidt, Dion Bajerski, Alicia S. Bicu, Victor Siefert, Miriam Eckl, Marvin Willam, Matthias F. Froelich, Stefan O. Schoenberg, Michael Ehmann, Daniel Buergy, Florian Stieler, Jens Fleckenstein, Frank A. Giordano, Judit Boda-Heggemann, Constantin Dreher

**Affiliations:** 1 Department of Radiation Oncology, University Medical Center Mannheim, Medical Faculty Mannheim, Heidelberg University, Theodor-Kutzer-Ufer 1-3, 68167 Mannheim, Germany; 2 DKFZ-Hector Cancer Institute, University Medical Center Mannheim, Mannheim, Germany; 3 Mannheim Institute for Intelligent Systems in Medicine (MIiSM), University of Heidelberg, Heidelberg, Germany; 4 Junior Research Group “Intelligent Imaging for adaptive Radiotherapy”, Mannheim Institute for Intelligent Systems in Medicine (MIiSM), University of Heidelberg, Heidelberg, Germany; 5 Department of Radiology and Nuclear Medicine, University Medical Center Mannheim, Medical Faculty Mannheim, Heidelberg University, Mannheim, Germany; 6 Junior Research Group “Image and Surface guided Radiotherapy”, Mannheim Institute for Intelligent Systems in Medicine (MIiSM), University of Heidelberg, Heidelberg, Germany

**Keywords:** Radiomics, Autocontouring, Adaptive radiotherapy, HyperSight-CBCT, Prostate cancer, Ethos, Cancer, Computational biology and bioinformatics, Medical research, Oncology, Urology

## Abstract

This study evaluated segmentation accuracy, efficiency, and radiomic feature stability for manual (MD), artificial intelligence-based (AI), and hybrid (MD + AI) contouring of pelvic organs on planning CT (pCT) and HyperSight cone-beam CT (hCBCT) for adaptive radiotherapy. Dice similarity and 95th percentile Hausdorff distance (HD95) quantified segmentation agreement, while radiomic feature stability was assessed using the concordance correlation coefficient (CCC). Agreement between segmentation approaches was highest for bladder and femora (median Dice 0.95–0.96; HD95 1.88–2.17 mm), intermediate for prostate and rectum (median Dice 0.92; HD95 2.22–2.62 mm), and lowest for seminal vesicles and penile bulb (median Dice 0.76–0.83; HD95 3.01–3.41 mm). AI and MD + AI reduced contouring times by about 90% and 60% compared to MD. Radiomic feature stability differed significantly between segmentation modes (all p_adj_ ≤ 0.05). GLRLM features exhibited significantly higher stability than other features, whereas morphological features showed lower stability. Median radiomic feature stability was highest for bladder and femora, and intermediate for prostate and rectum. In conclusion, AI-based and hybrid contouring achieved high accuracy and substantial time savings, while texture- and intensity-based radiomic features showed robustness with AI segmentation. This study demonstrated feasibility of extracting distinct, reliable quantitative parameters based on AI-only contouring of pelvic structures.

## Introduction

Accurate delineation of target volumes and organs at risk is a crucial component of modern radiotherapy, particularly within adaptive treatment strategies^1,2^. With the advent of artificial intelligence (AI) and advanced automated contouring systems such as recently introduced for the adaptive radiotherapy (aRT)-capable Ethos linear accelerator (Varian, Siemens Healthineers; eLinac), there is a growing potential and need to further reduce the time and personnel demands associated with manual segmentation by employing automated, AI-assisted approaches, especially in the field of high-precision adaptive radiotherapy^3,4^. These algorithms are applicable to both planning computed tomography (CT) scans and pre-fractional cone-beam CT (CBCT) scans, which are routinely acquired within CT-based aRT workflows^5,6^. Despite their clinical promise, automatically AI-generated contours can vary significantly in terms of their acceptance and accuracy, thus requiring physician-based correction. To assess the possibility of increased efficacy during aRT workflows and identify the full potential of autocontouring, the AI-only autocontouring mode must be compared to conventional, expert-drawn manual contours (MD) and to AI-based contours refined by clinician review according to the aRT workflow^4,7^. This is particularly relevant for autocontouring of the pelvic region, as robust, reproducible and time-efficient contouring may allow for accurate target and risk volume definition even though different fillings of hollow organs such as the bladder or rectum lead to high variability within this region. While conventional radiotherapy concepts only rely on contouring of the planning CT (pCT), aRT capable concepts further need contouring of fractional imaging. Although pCT imaging at a diagnostic CT scanner is currently the gold standard for contouring in treatment planning, the newly introduced fractional HyperSight-CBCT (hCBCT) imaging can also provide high-quality CT-based imaging with optimized tissue visualization and stable Hounsfield Units (HUs)^6–11^. For this reason, the true potential of AI-guided automated organ contouring must be assessed qualitatively and quantitatively, for both diagnostic CT scans and hCBCT scans, as applicable for high-precision aRT.

Based on the provided contouring during treatment planning, quantitative imaging parameters can be derived and assessed e.g. by the extraction of advanced radiomic features^12–15^. These features provide additional insights into tissue morphology and heterogeneity. They may even allow for the automated analysis of predictive radiological biomarkers for response and toxicity^16–18^.

Consequently, stable, automated, AI-based segmentation of pCT and hCBCT scans during high-precision radiotherapy at the eLinac may provide valuable radiological markers throughout the treatment course while saving time compared to manual contouring. This could facilitate structured, automated monitoring and targeting of these parameters^3,19^.

This study aims to systematically analyze the differences between manual and AI-guided automated contouring approaches for pelvic organs in patients treated with hCBCT-based radiotherapy for primary prostate cancer at the eLinac. The goal is to comprehensively assess the quality and efficiency of automated contouring solutions in the hCBCT-based adaptive workflow on the eLinac^3,4,10,12,13^, which could pave the way for AI-based autocontouring in radiological marker assessments.

## Materials and methods

### Study design and patients

Between October 2024 and April 2025, 50 patients with primary prostate cancer (Table 1) without metal hip implants undergoing definitive radiotherapy were prospectively enrolled in a registry trial set up for RT at the eLinac. All participants provided written informed consent prior to study enrollment. In cases where required, consent was obtained from the participant’s legal guardian(s). Ethical approval for this study was obtained from the Institutional Ethics Committee (reference number 2024_404 M-§ 47(3) MPDG), and all procedures were conducted as per relevant guidelines and regulations. Patient characteristics are summarized in Table 1.

**Table 1:** Baseline characteristics of the study cohort of patients with primary prostate cancer treated at HyperSight-CBCT equipped Ethos linear accelerator.

Patient characteristics	*n* (%) / Mean ± SD
Patients	50
Age (years)	
Mean ± SD	72.0 ± 8.2
Gleason score	
6	7 (14%)
7 (3 + 4)	26 (52%)
7 (4 + 3)	11 (22%)
8–10	6 (12%)
Tumor stage	
cT1	34 (68%)
cT2	10 (20%)
cT3	6 (12%)
Initial PSA [ng/ml]	
Mean ± SD	15.2 ± 25.3
Risk group according to D’amico^20^	
Low	5 (10%)
Intermediate	27 (54%)
High	18 (36%)
Dose concept of radiotherapy	
Ultra-hypofractionated	35 (70%)
Moderate Hypofractionated	8 (16%)
Mild hypo-/ normofractionated	7 (14%)

Data are presented as absolute numbers with percentages in parentheses, unless otherwise indicated. Age and prostate-specific antigen (PSA) values are shown as mean ± standard deviation (SD). Risk classification is according to d’Amico criteria^20^. Dose concepts include hypofractionated, moderate and mild hypo/normofractionated radiotherapy.

### Treatment planning and hCBCT imaging

**Fig. 1:**
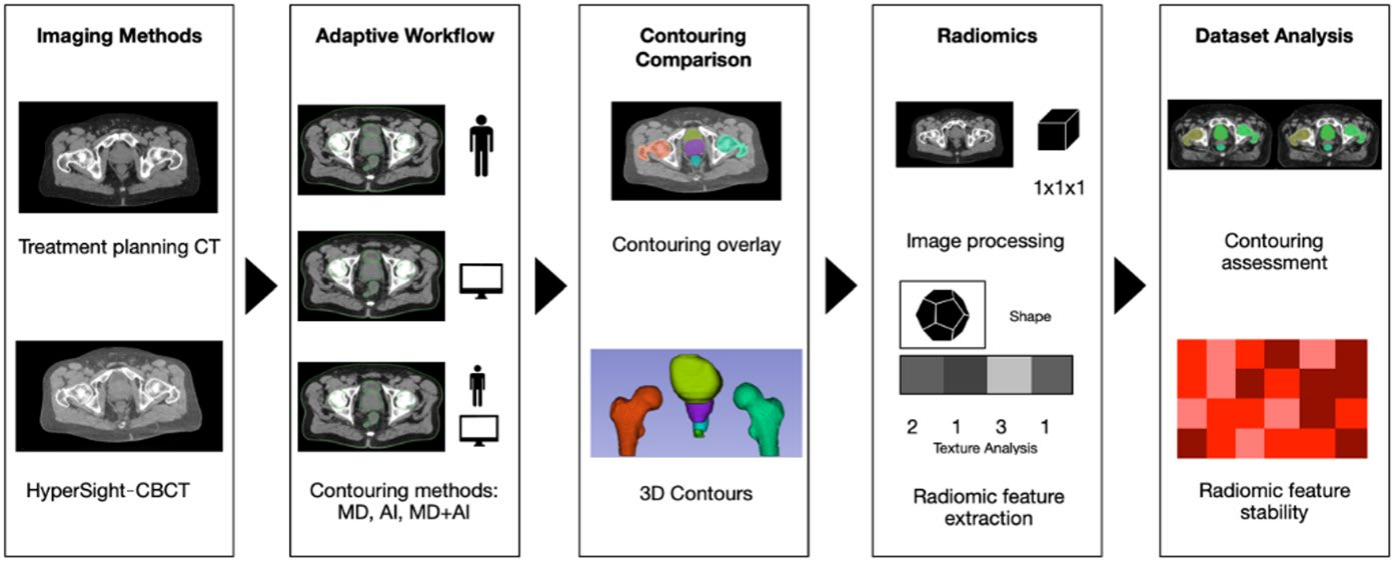
Study workflow of different contouring methods and quantitative imaging parameters assessment for patients with high-precision radiotherapy for prostate cancer. The imaging for radiotherapy at the Ethos linear accelerator (Varian, Siemens Healthineers) is based on a treatment planning CT (computed tomography) and fractional HyperSight-CBCT scans. Within the study workflow, the segmentation is performed within the Ethos Appliance Software, allowing for autocontouring by means of artificial intelligence-based (AI-only based) contouring (AI), or manual, clinician-drawn contouring (MD), and AI-based contouring with MD-based refinement (MD + AI). Based on these contouring methods the following assessments are performed: Segmentation comparisons and stability of quantitative imaging parameters by means of radiomic features.

RT was applied using the ring-based eLinac (Ethos, Varian, Siemens Healthineers). Treatment planning utilized the Ethos Appliance System and was based on a pCT scan (Brilliance BigBore, Philips, Netherlands) of the pelvic region (Fig. 1). The hCBCT imaging prior to each fraction of RT was performed using the preset “Pelvis” and the “iCBCT Acuros” reconstruction mode was employed^6^. The specific characteristics of the treatment planning CT scan and hCBCT “Pelvis” protocol are displayed in Table 2.

**Table 2:** Details of imaging protocols used for clinical application on pCT (planning computed tomography (CT) scanner: Brilliance BigBore, Philips) and HyperSight-CBCT Imaging (pelvis protocol) with iCBCT Acuros reconstruction mode at the Ethos linear accelerator (Varian, Siemens Healthineers).

Details of imaging protocols					
Imaging mode	kVp	mAs (mean ± SD)	Slice thickness (mm)	Pixel size (mm)	CTDIvol (mGy) (mean ± SD)
pCT	120	210.9 ± 55.2	2	1.2 × 1.2	12.5 ± 3.3
HyperSight-CBCT	125	466.6 ± 5.8	2	1.1 × 1.1	8.9 ± 0.1

Displayed are parameters for Peak kilovoltage (kVp), Milliampere-seconds (mAs), Slice thickness, Pixel size and CT dose index-volume (CTDIvol). For mAs and CTDIvol the mean value±standard deviation (SD) is displayed.

### Imaging analysis and radiomic features extraction

The pCT scan and the first hCBCT scan before the first fraction of RT were included and analyzed within the Ethos Appliance System for each of the 50 patients, summing up to 100 CT/CBCT scans analyzed in this simulation study. Three contouring methods for adaptive radiotherapy of prostate cancer were simulated and evaluated at the Ethos Appliance System for the following pelvic structures: prostate, seminal vesicles, bladder, penile bulb, rectum, right femoral head, and left femoral head. The three evaluated approaches comprised fully automated AI-only contouring (AI), the clinically semi-automated segmentation workflow of adaptive radiotherapy at the eLinac by means of AI-only based autocontouring with correction and validation by a physician (AI + MD), and the physician-only manual contouring (MD). Half of the cohort (*n* = 25) was segmented or validated by a senior physician with 9 years of experience in abdominopelvic CT imaging (R1), the other half by an experienced resident with 5 years of experience in abdominopelvic CT imaging (R2). For interobserver variability analysis, a subset of 20% of cases (*n* = 10) was independently contoured by both observers R1 and R2. The interobserver analysis was designed as an exploratory assessment of observer-related variability. All delineations were performed in a blinded manner. For each contouring method and each case, the required time for segmentation was prospectively recorded. Further, the time required for contour segmentation per slice was calculated (segmentation time/number of contoured slices). Figure 1 shows the workflow of adaptive radiotherapy and radiomics feature extraction.

The summarized analysis for (1) the interobserver subcohort (*n* = 10) and (2) the complete cohort (*n* = 50) was conducted in the two parts A, and B.

#### Part A: segmentation analysis and time assessment

The resulting segmentations were subsequently exported and analyzed using MATLAB R2024b (The MathWorks; Natick, MA, USA) for three-dimensional (3D) evaluation of geometric accuracy and volumetric agreement. For each patient, differences between contouring methods (MD, AI, and AI + MD) were quantified separately for pCT and hCBCT scans. The following geometric metrics were calculated: Dice Similarity Coefficient (DSC), 95th percentile Hausdorff distance (HD95) and 3D deviations of the center of mass (COM). COM deviation was quantified as the Euclidean distance between the COM coordinates of two segmentations, computed as

${\mathrm{COM}}=\sqrt {({\mathrm{x}}1 - {\mathrm{x}}{2^2})+{{\left( {{\mathrm{y}}1 - {\mathrm{y}}2} \right)}^2}+{{\left( {{\mathrm{z}}1 - {\mathrm{z}}2} \right)}^2}}$ and exported as a single scalar value for all statistical analyses^21^.

Comparisons were performed within modalities (separated for pCT or hCBCT) and between modalities (pCT vs. hCBCT). This allowed for an assessment of variability between methods and modalities. Segmentation times were compared accordingly, considering both total time and time normalized per contoured slice.

#### Part B: radiomic feature analysis

Whole-volume-of-interest (VOI) segmentations from pCT and hCBCT scans were processed as described previously^19^, using the open-source software LIFEx (version 25.06.1, https://www.lifexsoft.org). LIFEx provides a standardized pipeline for image preprocessing, feature calculation, and feature extraction, ensuring stability across different imaging modalities^22^. Preprocessing steps were performed uniformly across all scans and included spatial resampling to an isotropic voxel size of 1 × 1 × 1 mm^3^, intensity rescaling within a range of -1000 to 3000 HU and discretization of grey levels into 400 bins. These steps were chosen to harmonize input data, minimize variability introduced by acquisition parameters, and comply with the Image Biomarker Standardization Initiative (IBSI) guidelines^23^. A total of 112 IBSI-conformal radiomic features were extracted across seven feature groups: morphological, intensity-based, histogram-based, grey-level co-occurrence matrix (GLCM), grey-level run-length matrix (GLRLM), neighborhood grey-tone difference matrix (NGTDM), and grey-level size-zone matrix (GLSZM). These features formed the basis for subsequent stability analyses across segmentation methods for both imaging modalities.

### Statistical analysis

All statistical analyses were performed using R (version 4.2.1; R Foundation for Statistical Computing, Vienna, Austria).

For segmentation analysis, the geometric metrics were evaluated separately for pCT, hCBCT, and pooled across modalities. DSC values were classified as very high for > 0.9, high for 0.75–0.9, moderate for 0.5–0.74, and low for < 0.5. Differences between pCT and hCBCT were tested using paired Wilcoxon signed-rank tests and linear mixed-effects models.

Segmentation times were compared between contouring modes using Friedman tests with Bonferroni-adjusted post hoc Wilcoxon signed-rank tests. Percentage time savings relative to MD were additionally calculated.

In the overlap cohort, observer-specific comparisons (R1 vs. R2) were assessed separately using paired Wilcoxon tests. Assuming a two-sided paired design (α = 0.05, power = 80%), this sample size allows detection of large interobserver effects, corresponding to a minimum detectable effect size of Cohen’s d = 1.0.

Concordance correlation coefficients (CCC) were calculated to assess radiomic feature stability between the contouring methods.

The analysis included an interobserver analysis of the overlap cohort (R1 vs. R2) and an intermethod analysis of the three contouring approaches (MD, MD + AI, AI) across all volumes of interest (VOIs), including bladder, rectum, prostate, seminal vesicles, penile bulb, and left and right femora. CCC values were classified as very high for > 0.9, high for 0.75–0.9, moderate for 0.5–0.74, and low for < 0.5^24^.

CCC were compared across feature groups and modalities and visualized using heatmaps and boxplots.

Further, a linear mixed-effects model with contouring approach, feature group, and imaging modality as fixed effects, and random intercepts for patient and feature to account for within-patient correlations and inter-feature dependencies was used. To quantify potential volume dependency of radiomic stability, log-transformed contour volume was incorporated as a fixed effect in the mixed-effects model, including an interaction term with feature group.

The level of statistical significance was defined as p_adj _≤ 0.05 with post-hoc adjustment by Bonferroni for all tests.

## Results

### Part A: interobserver contouring assessment

#### A: Interobserver segmentation stability

Across the overlap cohort (pooled for pCT and hCBCT), the interobserver agreement of MD contours, as measured by the DSC coefficient, was very high for the bladder (0.96), left femur (0.97), and right femur (0.96); high for the rectum (0.91), seminal vesicles (0.86), and prostate (0.85); and moderate for the penile bulb (0.59).

Pooled median interobserver COM deviations for MD contours were for the bladder 0.55 mm, left femur 0.69 mm, right femur 0.77 mm, rectum 0.99 mm, prostate 1.27 mm, penile bulb 1.36 mm, and seminal vesicles 1.39 mm.

Pooled HD95 values for MD contours were for the bladder 2.1 mm, left femur 1.87 mm, right femur 3.1 mm, rectum 2.2 mm, prostate 2.3 mm, seminal vesicles 2.3 mm, and penile bulb 3.4 mm.

MD + AI contours demonstrated significantly higher interobserver DSC than MD contours for the bladder, both femora, prostate, and seminal vesicles on both pCT and hCBCT scans (all p_adj_ ≤ 0.05).

For COM, significantly lower values were observed for MD + AI compared with MD for the bladder and both femora on pCT, whereas on hCBCT, a significant reduction was observed for the prostate only (all p_adj_ ≤ 0.05).

For HD95 values, significantly lower values were observed for the bladder and femora for MD + AI compared with MD in both modalities (all p_adj_ ≤ 0.05). For the prostate and seminal vesicles, HD95 values were significantly lower for MD + AI compared with MD in hCBCT (p_adj_ ≤ 0.05).

#### Interobserver analysis of segmentation time

In the overlap cohort, no significant differences in segmentation times between R1 and R2 were observed. This held true for both pooled data and data stratified by modality, as well as for total times and per-slice estimates (all p_adj_ > 0.05). Interobserver time differences exhibited substantial variability, with means±standard deviations (SD) of -171.7 ± 97.6 s (pCT, MD), -83.2 ± 85.3 s (pCT, MD + AI), -161 ± 147.8 s (hCBCT, MD), and − 101.2 ± 70.1 s (hCBCT, MD + AI). Based on the observed variability, the minimum detectable interobserver difference ranged from approximately 70 to 150 s (assuming Cohen’s d = 1.0).

### B: Interobserver stability of radiomic features

Across all structures, the stability of radiomic features between observers was consistently very high.

For MD, the pooled median CCC across all radiomic features and organs was 0.99, with comparable results for pCT (0.99) and hCBCT (0.99).

For MD + AI contours, the pooled median CCC was 0.99 across all features and organs, with comparable stability for pCT (0.99) and hCBCT (0.99).

At the organ level, both MD and MD + AI contours demonstrated very high stability for the bladder, rectum, prostate, and femora (with a median CCC > 0.98 across modalities).

The seminal vesicles showed slightly lower stability, with a median CCC = 0.88 for MD, and 0.92 for MD + AI. The penile bulb exhibited the lowest stability, with a median CCC = 0.76 for MD, and 0.89 for MD + AI.

Directly comparing interobserver CCC values between MD and MD + AI contours revealed no significant differences in either modality or in the pooled analysis (all p_adj_ > 0.05).

### General contouring assessment

#### A: Segmentation stability

**Fig. 2:**
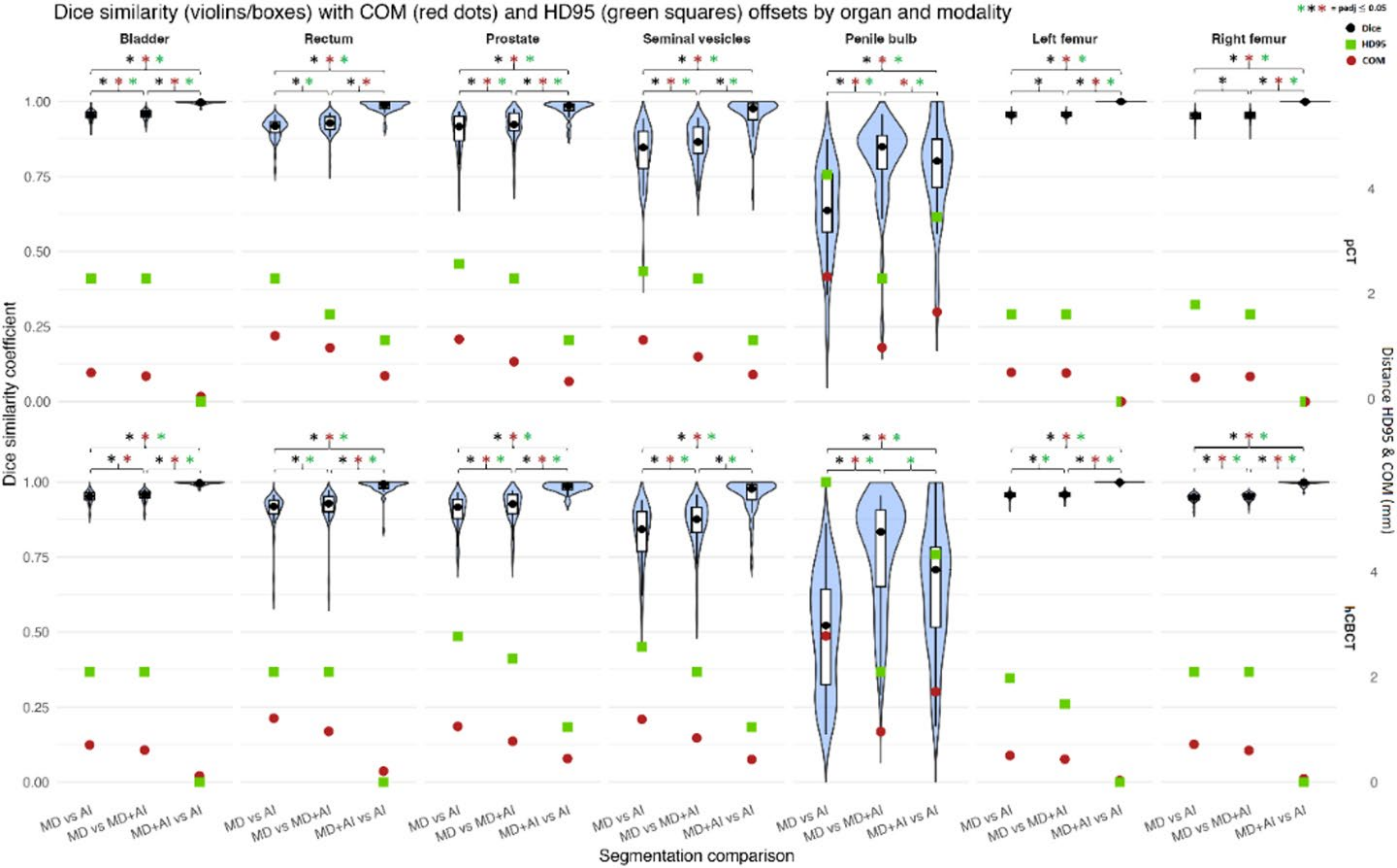
Violin plots of Dice similarity coefficients (left y-axis) and overlaid median center-of-mass (COM) shifts and 95th-percentile Hausdorff distances (HD95) in millimeters (right y-axis) across pelvic organs and segmentation pairings, shown separately for planning CT (pCT) and HyperSight-CBCT (hCBCT). Each violin displays the distribution of patient-level Dice values, with boxes indicating interquartile ranges and black dots denoting medians. Median COM deviations are shown as red dots, while median HD95 values are displayed as green squares. Pairings are defined as MD (manual delineation), AI (automatic segmentation), and MD + AI (AI segmentation with manual correction). Significant differences are marked with asterisks (black for Dice values, red for COM values and green for HD95).

A comparison of DSC between segmentation mode pairings (MD vs. AI, MD vs. MD + AI, MD + AI vs. AI) within each organ and modality demonstrated the lowest median DSC values for the MD vs. AI pairing (p_adj_ ≤ 0.05) (Fig. 2). Consistent results were observed for HD95, with the MD vs. AI pairing showing significantly larger boundary deviations for the bladder, rectum, prostate, and seminal vesicles on both pCT and hCBCT (p_adj_ ≤ 0.05).

For the penile bulb, the DSC values of MD vs. MD + AI compared to MD + AI vs. AI were not significantly different on both pCT and hCBCT (p_adj_ > 0.05). For HD95, all segmentation mode pairings were significantly different for the penile bulb on both pCT and hCBCT (all p_adj_ ≤ 0.05). For the left and right femora, all DSC-based segmentation mode pairings were significantly different on both pCT and hCBCT (all p_adj_ ≤ 0.05). For HD95, a significant difference between MD vs. MD + AI and MD + AI vs. AI was observed for both femora on pCT, whereas the comparison between MD vs. AI and MD vs. MD + AI was not significant. On hCBCT, all segmentation mode pairings differed significantly (all p_adj_ ≤ 0.05).

For the comparison of DSC between pCT and hCBCT for identical segmentation mode pairings, significant modality effects were only present for the penile bulb, with higher DSC values on pCT than on hCBCT for both MD vs. AI and MD + AI vs. AI (p_adj_ ≤ 0.05), and for the femora, with significant higher values on pCT for the MD vs. AI pairing (p_adj _≤ 0.05). In contrast, HD95 did not reveal any significant modality-dependent differences between pCT and hCBCT for any organ or segmentation pairing after correction for multiple testing (p_adj_ > 0.05).

**Fig. 3:**
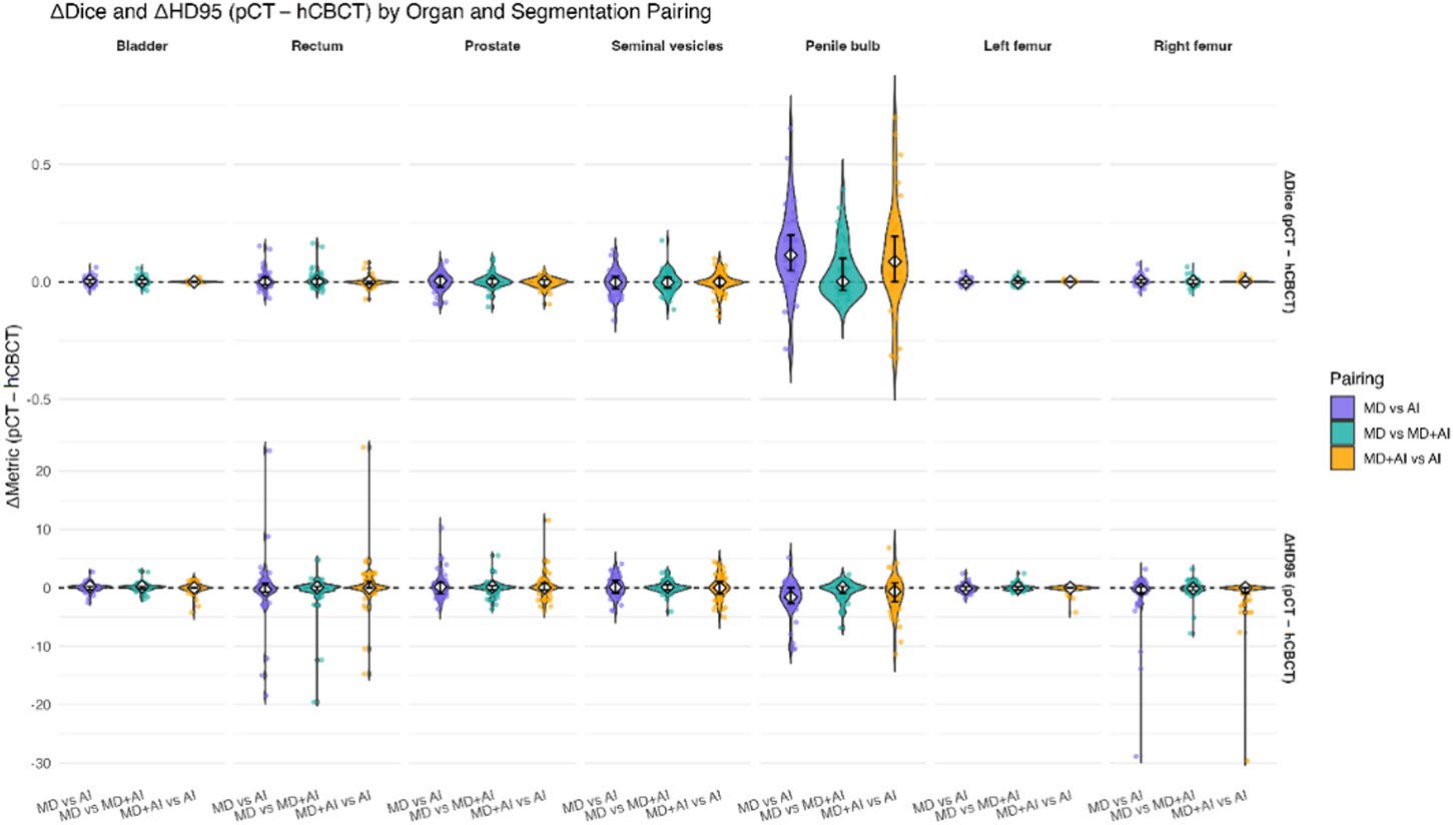
Violin plots of patient-level Dice and 95th-percentile Hausdorff distances (HD95), (ΔDice, ΔHD95, planning CT [pCT] minus HyperSight cone-beam CT [hCBCT]) across pelvic organs and segmentation pairings. Violins show the distribution of ΔDice (top row) and ΔHD95 (bottom row) values; individual points represent patients; white diamonds indicate medians; and vertical bars denote the interquartile range (IQR). Pairings are defined as MD (manual delineation), AI (automatic segmentation), and MD + AI (AI segmentation with manual correction). Positive Δvalues indicate higher Dice or HD95 on pCT, negative values indicate higher Dice or HD95 on CBCT.

The corresponding ΔDSC analysis (pCT-hCBCT) (Fig. 3) revealed that differences between segmentation mode pairs persisted as positive ΔDSC values but did not reach statistical significance in the comparison between segmentation mode pairs (p_adj_ > 0.05). A comparable pattern was observed for ΔHD95, with no significant differences between segmentation mode pairings (p_adj_ > 0.05).

Regarding the COM deviations, significant differences were less frequent compared to the DSC value comparisons as highlighted in Fig. 2. The highest COM deviation values were present in the MD vs. AI pairing of segmentation modes for all organs and both scan modalities.

Comparing COM deviations between pCT and hCBCT for identical segmentation mode pairings showed significant modality effects only for both femora for the MD vs. AI and MD + AI vs. AI comparisons (p_adj_ ≤ 0.05).

Across organs, segmentation accuracy differed significantly for DSC, HD95 and COM metrics (all p_adj_ ≤ 0.05). For the MD vs. AI comparison, DSC was significantly higher for the bladder and femora than for all other organs (p_adj_ ≤ 0.05). The seminal vesicles and penile bulb showed the lowest DSC values (p_adj_ ≤ 0.05 for the comparison with bladder and femora). This pattern was consistent across pCT and hCBCT.

HD95 values differed significantly across organs on both pCT and hCBCT. Both femora demonstrated the lowest HD95 values and were significantly lower than the rectum, prostate, seminal vesicles, and penile bulb on both modalities (all p_adj_ ≤ 0.05). On pCT, femoral HD95 values were additionally lower than bladder values (p_adj_ ≤ 0.05).

COM deviations differed significantly across organs on both pCT and hCBCT. Both femora demonstrated the lowest COM differences and were significantly lower than the rectum, seminal vesicles, and penile bulb on both modalities (all p_adj_ ≤ 0.05). On hCBCT, femoral COM deviations were additionally lower than prostate values (p_adj_ ≤ 0.05). The bladder showed significantly lower COM differences than the rectum, prostate, seminal vesicles, and penile bulb on both modalities (all p_adj_ ≤ 0.05). The penile bulb showed the greatest COM deviations, which were significantly higher than those of the bladder and femoral contours in both modalities (all p_adj_ ≤ 0.05).

A similar pattern was observed for MD vs. MD + AI, with bladder and femora again achieving the highest DSC compared to all other organs and seminal vesicles and penile bulb showing significantly lower DSC values compared to bladder and femora (each p_adj_ ≤ 0.05).

HD95 values also differed significantly across organs on both pCT and hCBCT. The femora demonstrated the lowest HD95 values and were significantly lower than the rectum, prostate, seminal vesicles, and penile bulb on both modalities (all p_adj_ ≤ 0.05). On pCT, femoral HD95 values were lower than prostate and seminal vesicle values (all p_adj_ ≤ 0.05). The bladder showed significantly lower HD95 values than the rectum, prostate, seminal vesicles, and penile bulb on both modalities (all p_adj_ ≤ 0.05).

For COM deviations, the bladder and femora exhibited the lowest values (p_adj_ ≤ 0.05 for the comparison with all other organs), while the penile bulb and seminal vesicles showed the largest shifts (p_adj_ ≤ 0.05 for the comparison with bladder and femora). This pattern was consistent across pCT and hCBCT.

### Analysis of segmentation time

Segmentation time differed significantly across segmentation modes in both pCT and hCBCT, as well as in the pooled analysis (all p_adj_ ≤ 0.05). Post-hoc comparisons confirmed that both AI and AI + MD segmentations were significantly faster than MD (p_adj_ ≤ 0.05) across pCT, hCBCT, and in the pooled analysis, whereas AI and AI + MD did not differ significantly from each other. These findings were consistent for total times and per-slice estimates.

**Fig. 4:**
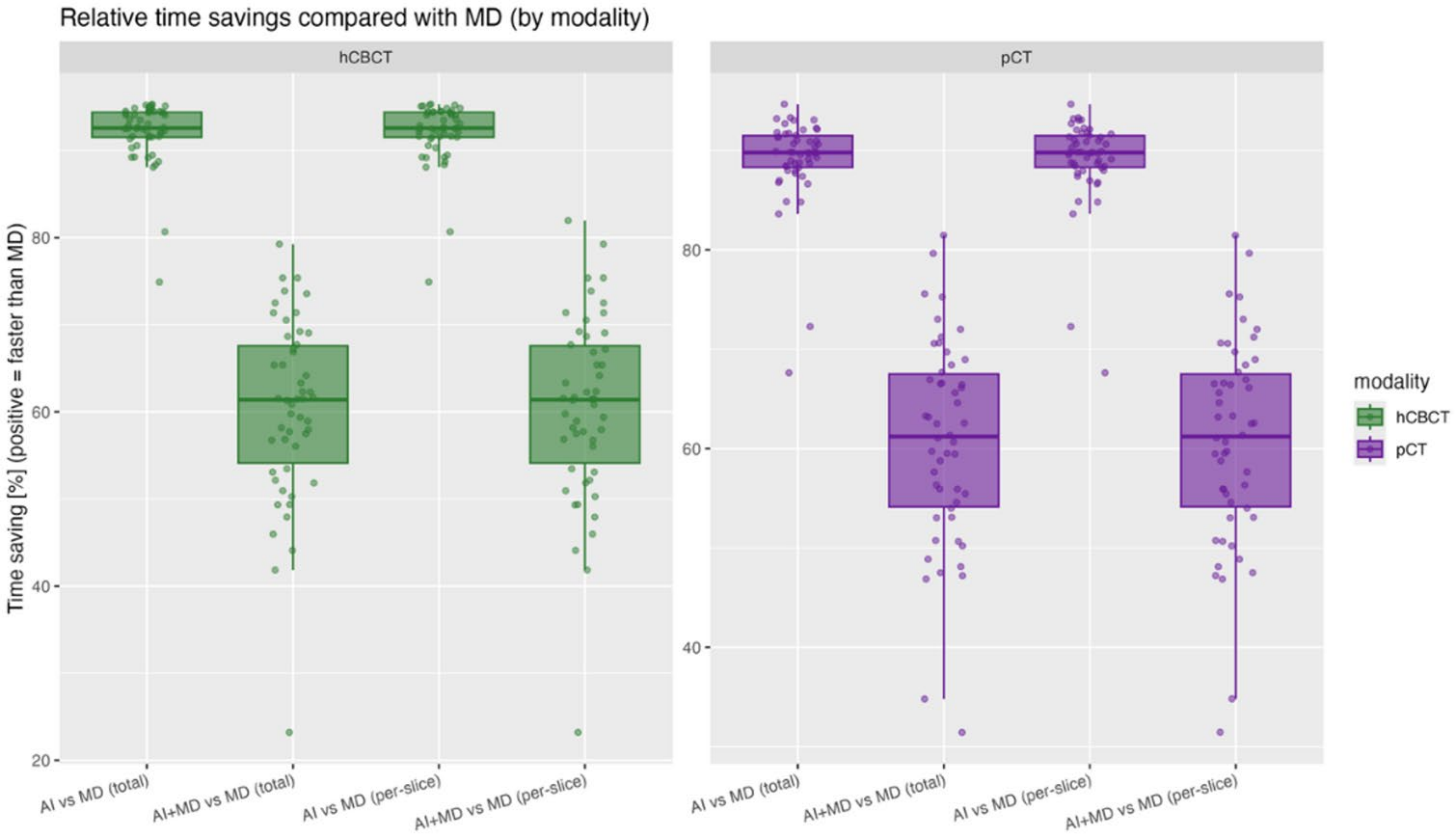
Relative contouring time savings by modality and segmentation mode. Box-and-jitter plots show percent time saving of AI-only autocontouring (AI) and AI-based autocontouring with physician-based validation (AI + MD) compared to manual delineation (MD), displayed for both total time and time per contoured slice, and faceted by modality (planning CT (pCT, purple colours) and HyperSight-CBCT (hCBCT, green colours)). Boxes represent interquartile range (IQR) with median lines; whiskers extend to 1.5×IQR; points are patient-level values (median per patient/mode). Higher positive percentage values indicate faster performance than MD.

AI decreased the segmentation time by over 90%, across both modalities, while AI + MD reduced times by about 60% compared to MD. These patterns remained consistent when expressed per slice and when stratified by pCT versus hCBCT (Fig. 4).

When comparing modalities (pCT vs. CBCT), segmentation times did not differ significantly within any segmentation mode (MD, AI, MD + AI), consistent for both total time and per-slice estimates (all p_adj_ > 0.05).

#### B: Stability of radiomic features (Fig. 5)

The mixed-effects analysis demonstrated a significant main effect of segmentation approach comparison on radiomic stability across pCT, hCBCT, and the pooled dataset (all *p* < 0.05).

Post-hoc tests derived from the mixed-effects model showed that radiomic stability for AI vs. MD, while high in absolute terms, was significantly lower (median CCC = 0.93 [pCT 0.93, hCBCT 0.92]) than for MD + AI vs. MD (median CCC = 0.94 [pCT 0.94, hCBCT 0.94]) and AI vs. MD + AI (median CCC = 0.94 [pCT 0.94, hCBCT 0.94]) (all p_adj_ ≤ 0.05). Within organs, no significant difference was observed between MD + AI vs. MD and AI vs. MD + AI (all p_adj_ > 0.05). Separate mixed-effects models were additionally fitted for each segmentation pairing. These analyses confirmed a significant organ effect across all pairings but did not identify additional effects beyond those already captured by the significant comparison-by-organ interaction in the full model (all p_adj_ > 0.05). A significant main effect of imaging modality on radiomic stability was also observed in the mixed-effects model (*p* < 0.05), with slightly higher stability estimates on hCBCT than on pCT. However, modality-specific post-hoc contrasts did not reveal statistically significant differences between pCT and hCBCT after correction for multiple testing (all p_adj_ > 0.05).

##### Radiomic feature group effects

A significant overall effect of radiomic feature group on radiomic stability was observed in the mixed-effects model (*p* < 0.05).

**Fig. 5:**
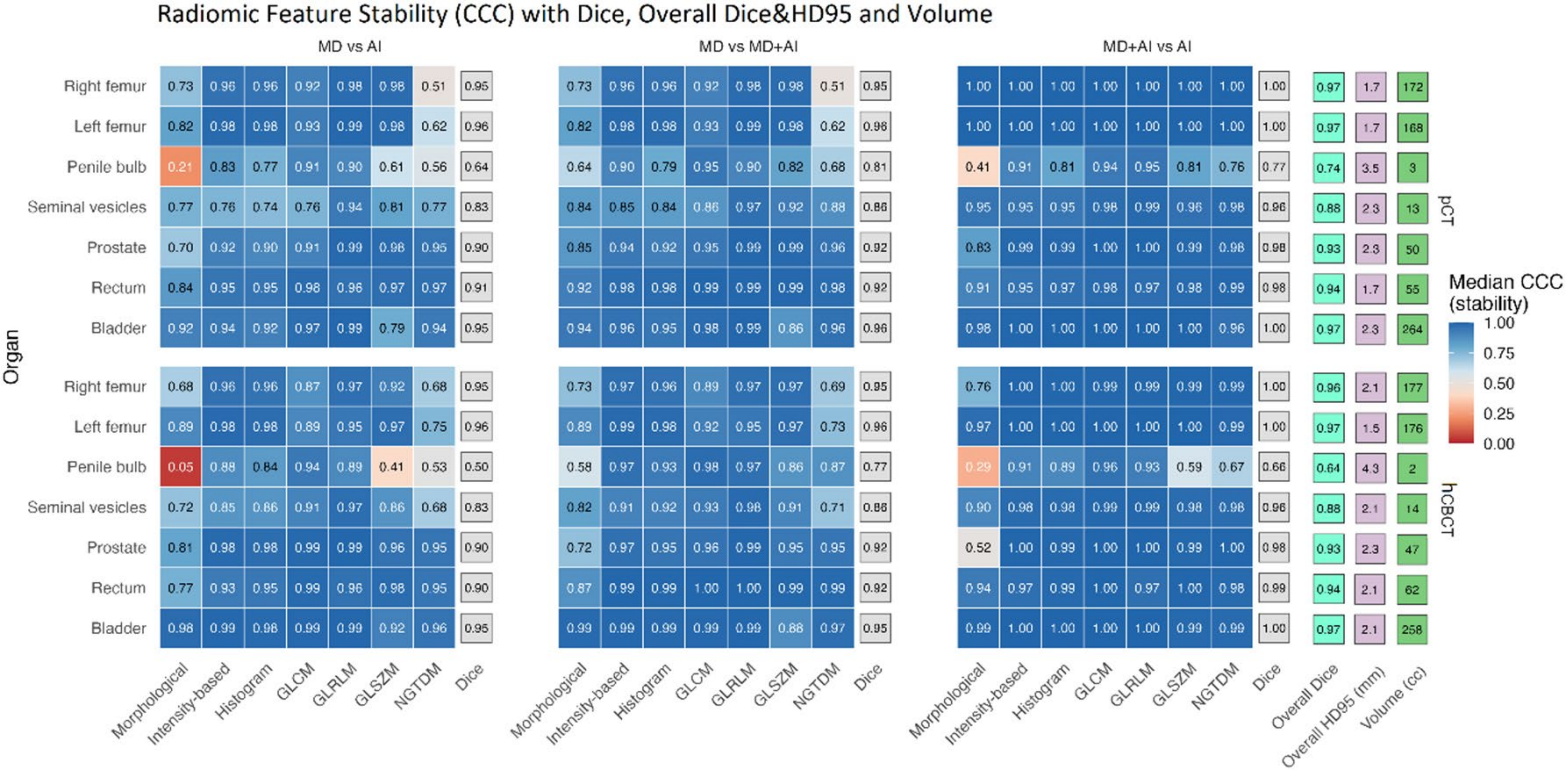
Radiomic feature stability by organ, feature group, modality, and contour pairing, including geometric overlap (Dice) and 95th percentile Hausdorff distance (HD95). Heatmap tiles display the median concordance correlation coefficient (CCC) for radiomic features, summarized by organ (rows) and feature group (columns). Panels are faceted by modality (planning CT (pCT) vs. HyperSight-CBCT (hCBCT)) and by segmentation comparison (MD vs. AI, MD vs. MD + AI, MD + AI vs. AI).

GLRLM features showed the highest stability estimates across modalities (median CCC: 0.97 [pCT], 0.97 [hCBCT]). In post-hoc analysis, GLRLM features exhibited significantly higher stability than both NGTDM (median CCC: 0.90 [pCT], 0.91 [hCBCT]), and intensity-based features (median CCC: 0.96 [pCT], 0.97 [hCBCT]) (p_adj_ ≤ 0.05). Intensity-based features, GLCM (median CCC: 0.95 [pCT], 0.96 [hCBCT]), GLSZM (median CCC: 0.95 [pCT], 0.96 [hCBCT]), and Histogram features (median CCC: 0.94 [pCT], 0.96 [hCBCT]) demonstrated higher stability estimates than Morphological (median CCC: 0.92 [pCT], 0.93 [hCBCT]) and NGTDM features, although these differences did not reach statistical significance after correction for multiple testing (all p_adj_ > 0.05). See Fig. 5 for reference.

##### Organ-specific effects

The mixed-effects model revealed a significant main effect of organs on radiomic feature stability (*p* < 0.05). Stability was significantly higher in large, high-contrast organs, including the bladder, prostate, and femora, compared with small or low-contrast structures, particularly the seminal vesicles and penile bulb (all p_adj_ ≤ 0.05).

The interaction between organ and segmentation pairing was not statistically significant (*p* > 0.05). No significant modality-dependent differences in organ-specific radiomic stability were detected between pCT and hCBCT (*p* > 0.05).

The three most and least stable radiomic features per organ and modality are summarized in Table 3.

Contour volume was significantly associated with radiomic stability (*p* < 0.05), and the magnitude of this association differed significantly between feature groups (FeatureGroup × logVolume interaction, *p* < 0.05). Post-hoc comparisons indicated that morphological, intensity-based, and GLSZM features exhibited stronger volume dependency than GLCM and GLRLM features (all p_adj_ ≤ 0.05).

Within each facet, the light-grey “Dice” column indicates the median Dice coefficient for that specific contour pairing, while the “Overall Dice” column (aquamarine) summarizes the average Dice value across all three pairings for each organ and modality. The purple column summarizes the average HD95 values across all three pairings for each organ and modality. An additional “Volume (cc)” column reports the median segmented organ volumes. Numeric labels inside tiles report the median CCC for each feature group. Across organs, GLRLM features display the highest stability (deep blues), followed by GLCM/GLSZM and the Histogram/Intensity-based groups, while Morphological and NGTDM features tend to be less stable. Larger, well-defined structures such as the bladder and femora demonstrate both higher CCCs and Dice values, while smaller or low-contrast organs (e.g., penile bulb, seminal vesicles) exhibit reduced stability.

**Table 3:** Least and most stable radiomics features across pelvic organs for planning CT (pCT) and HyperSight-CBCT (hCBCT).

Organ	Most stable 3 features	Least stable 3 features	Most stable 3 features	Least stable 3 features
	pCT		hCBCT	
Bladder	IH_IQR	IH_MinGrey	IH_Mode	IB_CV
	IH_QCD	IB_Min	IH_GradMax	GLSZM_SmallZoneLowGLEmph
	IH_Median	IB_CV	IH_Median	GLSZM_NormZSNU
Penile bulb	GLCM_Dissimilarity	Comp2	GLCM_Contrast	IB_QCD
	GLCM_DiffAvg	Comp1	GLCM_DiffAvg	Sph_Disprop
	GLCM_DiffVar	IH_Kurtosis	GLCM_Dissimilarity	Asphericity
Left femur	IH_MaxGrey	IB_Min	IH_MaxGrey	GLSZM_SmallZoneLowGLEmph
	IB_Min	IH_MinGrey	IB_Max	GLRLM_ShortRunLowGLE
	IB_Max	NGTDM_Coarseness	GLSZM_LargeZoneEmp	GLRLM_LowGLEmph
Right femur	IH_GradMax	NGTDM_Coarseness	IB_Median	GLSZM_ZoneSizeNU
	IH_Range	NGTDM_Busyness	IB_P50	GLSZM_NormZSNU
	IH_MinGrey	IH_GradMin	IB_P10	GLSZM_SmallZoneLowGLEmph
Prostate	IH_Median	Sph_Disprop	IH_Mode	Sphericity
	IH_GradMax	Asphericity	IH_Median	Comp1
	IH_GradMin	Sphericity	IB_Max	Comp2
Rectum	IH_GradMin	IB_CV	IH_P90	IB_CV
	IB_Max	IB_QCD	IH_MinGrey	IH_QCD
	GLRLM_LongRunEmphasis	IB_RobustMAD	IB_Min	GLSZM_LargeZoneLowGLEmph
Seminal vesicles	GLCM_SumEntropy	IB_CV	GLCM_SumEntropy	IB_CV
	GLCM_DiffEntropy	IB_QCD	GLCM_DiffEntropy	IB_QCD
	GLCM_ClustTend	GLCM_ClustProminence	GLCM_ClustProm	GLSZM_SmallZoneEmp

For each organ, the three most stable features and the three least stable features are listed. Abbreviations: IH = Intensity Histogram; IB = Intensity-Based; GLCM = Grey-Level Co-occurrence Matrix; GLRLM = Grey-Level Run-Length Matrix; GLSZM = Grey-Level Size-Zone Matrix; NGTDM = Neighbourhood Grey-Tone Difference Matrix; Comp = Compactness; Sph_Disprop = Spherical Disproportion; MAD = Mean Absolute Deviation; NU = Non-Uniformity; NormZSNU = Normalised Zone Size Non-Uniformity.

## Discussion

Here, we present the first comprehensive analysis of the precision of AI-based contouring and the subsequent extraction of radiomic features for patients undergoing definitive radiotherapy for prostate cancer using an online aRT capable, hCBCT equipped eLinac. Contouring precision for large pelvic structures including the prostate itself was very high, especially when using AI-based contouring with physician-based validation. This method showed high radiomic feature stability in both hCBCT and diagnostic pCT. In contrast, for smaller or low-contrast structures such as the penile bulb and seminal vesicles, radiomic feature stability was reduced compared with larger organs. This finding must be interpreted in the context of the strong volume dependence of overlap-based metrics and radiomic features, whereby small absolute geometric deviations may disproportionately affect DSC and radiomic stability estimates.

Texture-based feature groups - particularly GLRLM and GLCM - demonstrated higher stability, with GLRLM features showing statistically significant advantages over selected feature groups.

Notably, even for the automated, AI-only contours which are characterized by the greatest time efficiency in contouring and extracting radiomic features, stable texture-derived features were retained in the prostate itself and other larger organs in both hCBCT and diagnostic pCT. However, small structures exhibited pronounced instability of radiomic features.

Together with the significant time efficiency observed in AI-assisted segmentation, these results imply that specific radiomic feature groups derived from automated AI-only-based contouring on hCBCT could already serve as a stable basis for extracting and monitoring quantitative imaging markers during adaptive radiotherapy. This could enable the automated, AI-only based targeting of radiomic markers, particularly those of the prostate, without physician-based validation during prostate cancer high-precision radiotherapy with hCBCT imaging.

The practical advantages observed in this study stem from two main aspects: (i) AI-assisted segmentation substantially reduces contouring time, and (ii) hCBCT provides CT-comparable radiomic stability, enabling robust radiomic features to be extracted directly from fractional hCBCT during high-precision radiotherapy.

For small, anatomically variable organs, reduced geometric agreement was observed for overlap-based metrics on both hCBCT and pCT. This finding is consistent with Sarria et al. who reported decreasing contouring stability with decreasing organ size^25^. In addition to the study by Sarria et al., we also included HD95 as a boundary-based metric that is less sensitive to volume effects, allowing us to differentiate between purely volume-driven variability and intrinsic organ-specific differences in geometric agreement^25^. Accordingly, a significant association was found between contour volume and radiomic stability. Post-hoc analyses demonstrated a stronger volume dependency of morphological, intensity-based, and GLSZM features compared with GLCM and GLRLM features. These findings provide a mechanistic explanation for the observed decrease in radiomic feature stability in smaller organs, particularly for shape- and intensity-derived metrics, which aligns with the partially demonstrated size dependency of radiomic feature stability in a phantom analysis by Jensen et al.^26^. Contrary to Yoon et al.‘s findings, our data shows texture-based features, particularly GLRLM, were among the most stable across contouring modes and modalities^27^. However, morphological features are less stable, though some individual morphological features are among the most stable radiomic features. These results highlight that texture features derived from high-quality hCBCT imaging can be highly stable for modern AI-assisted segmentation but must be critically evaluated for each feature‘s specific stability for each individual feature. However, our results indicate that segmentation-related variability is inherently low when automated radiomic features are derived from large, high-contrast organs, particularly for robust feature classes such as GLRLM.

The tendency toward increased interobserver variability for smaller organs could be reduced by applying a combined approach of AI-based contouring and physician-based validation. This approach would increase stability of delineations across all organs, reduce reader-to-reader dispersion and improve boundary consistency.

In our time analysis, AI-only based contouring reduced segmentation time per slice by about 90% compared to manual contouring, and the combined approach of AI-based and physician-validated contouring by about 60%, which is in line with similar studies of auto-contouring^28,29^. From a clinical standpoint, this magnitude is clinically highly relevant because it shortens the time required for the on-couch adaptive loop process time into a feasible treatment-slot window, reducing the delay between imaging and beam-on time^30,31^. Shorter turnaround limits inter- and intrafraction anatomical drift (e.g., bladder/rectum filling, or prostate motion), which in turn improves plan robustness and supports more accurate dose calculation at the moment of delivery^32^. The saved time can be reinvested in concurrent monitoring and quality assurance (e.g., verification imaging, motion checks) without prolonging sessions, and it makes adaptive re-optimization viable on more fractions and sites. Operationally, this efficiency frees staff capacity so more patients can receive time-intensive aRT on a given day, without sacrificing segmentation accuracy, improving treatment robustness and the accuracy of dose calculation on the re-optimized anatomy^33^. Future research should investigate the true potential of this time efficacy in automated segmentation, and how clinical decisions may profit from this time advantage, such as tailoring the adaptive treatment to potentially predictive, automatically, AI-based generated imaging markers (e.g. GLRLM-based radiomic features). Therefore, it is of high relevance, to investigate factors influencing the stability of automated contouring and the secondary derived radiomic features of these contours. Such robust features, if also demonstrated to be clinically predictive for response or toxicity, may serve as imaging targets for adaptive, high precision radiotherapy.

When comparing segmentation modes, the reduced agreement of AI-only contours in small and low-contrast organs was primarily reflected in overlap-based metrics. However, HD95 suggested that boundary deviations remained limited. Importantly, the MD + AI approach consistently mitigated these discrepancies, highlighting its added value especially in anatomically challenging structures.

Pure manual-based segmentations were significantly more time-consuming. This stepwise enhancement using a hybrid approach is clinically significant because it shows that artificial intelligence can provide a high-quality baseline, particularly in large and robust structures. Additionally, expert adjustments ensure stability in anatomically challenging structures, leading to reduced interobserver variability. Other contouring studies have shown that deep-learning–based autosegmentation can significantly reduce contouring time and interobserver variability. However, hybrid approaches offer the most balanced combination of efficiency and accuracy^34–36^. As an alternative to the investigated method of deep learning-based autocontouring within the Ethos Appliance Platform for adaptive radiotherapy, different approaches of autocontouring such as atlas-based or deformable registration based autocontouring platforms should be discussed as well. These platforms have high performance for stable geometry and bony landmarks, but they drop due to inter-/intrafraction variability, organ filling changes, and hCBCT artifacts which in turn hampers the time efficacy^36–38^. Correspondingly, deep learning-based approaches of autocontouring tend to be effective for large cohorts, but may also lose efficacy in case of domain shift^34^.

In addition to the discussed contouring analysis, the robustness and efficacy of the different segmentation methods must be translated into quantitative imaging assessments, such as radiomics, as investigated by this study. If feasible, the automated calculation of automatically segmented structures or targets in oncologic and radio-oncologic imaging could enable standardized monitoring and even adaptive treatment adjustments based on stable target markers. Radiomics, the in-depth quantitative analysis of medical imaging data, has become a major topic in clinical research, particularly in radiation oncology and oncologic imaging. Studies in this area have the potential to improve imaging-based decision-making by identifying imaging biomarkers, which could lead to personalized treatment options^14,15^. However, most radiomics research in prostate cancer patients has focused on MRI-based analyses^39–41^, studies from Tanadini-Lang et al., Osman et al., and Ching et al. already provide evidence that CT-based radiomic features may be of informative and predictive value in prostate cancer imaging and also longitudinal monitoring, such as by fractional hCBCT imaging during radiotherapy^41–45^. However, understanding of radiomic analysis as part of IGRT is still limited. Imaging with standard CBCT is usually associated with significantly reduced image quality and possible artefacts in routine clinical practice, which is why it is primarily used for visual imaging guidance^46–49^. Delgadillo et al. were able to demonstrate a high repeatability and even clinical prediction of toxicity of CBCT-based radiomic features (texture-derived (GLCM, GLSZM) and first-order intensity features in iCBCT reconstruction^50–52^. In our study, these overlapping feature classes showed significant stability in the AI-only setting, particularly for large, high-contrast organs such as the bladder and rectum. Moreover, Schmidt et al., who applied a different approach with deformable registration–based contour propagation of the prostate with fiducials across advanced hCBCT demonstrated excellent contour agreement metrics and found that the majority of extracted radiomic features provided high stability^11^. Another study by Cvachovec and Bicu et al. translated this finding into high-quality imaging by means of hCBCT imaging in patients undergoing adaptive SBRT for prostate cancer at the O-ring Ethos linear accelerator without any fiducials, and even demonstrated a correlation between the longitudinal progression of radiomic features and the fraction number of adaptive SBRT for the prostate^19^.

Building on prior work^19^, the present study extends the analysis to multiple pelvic organs using both pCT and hCBCT imaging, and various segmentation approaches, including AI-only contouring. By examining a cohort of prostate cancer patients without implanted gold fiducial markers, the study reflects clinical workflows that rely on imaging-based delineation rather than marker-guided localization. This enables broad application with translational potential for other areas of routine clinical practice.

Taken together, this supports that while AI-based segmentation can facilitate stable radiomic extraction, feature class and anatomical context critically determine stability of quantitative imaging for high precision radiotherapy^53^. This should be considered before evaluating AI-only radiomic features, especially regarding targeted adaptive radiotherapy, possibly taking into account automatically generated radiomic features during radiotherapy course.

Accordingly, this study demonstrates the feasibility of radiomic feature extraction from hCBCT-imaging within an adaptive radiotherapy workflow using AI-based and hybrid contouring approaches. The results highlight conditions under which stable radiomic features can be obtained across segmentation methods and imaging modalities. While these findings support the methodological robustness of automated AI-based radiomic analyses of hCBCT imaging scans, they do not establish clinical utility or predictive relevance. The clinical significance of such features must be investigated in future outcome-driven studies. However, the study paves the way for such investigations thus unlocking new possibilities for personalized treatment approaches in adaptive radiotherapy. The integration of hCBCT-based scans into high-precision radiation therapy represents a paradigm shift in radiation therapy, not only because of the high diagnostic-quality image quality^6,11^. The presented study should serve as a hypothesis-generating basis for future research on the clinical translation of automated image analysis in personalized radiotherapy approaches. Translational to MR-based dose escalation for local therapy intensification and use of MR-based biomarkers^54–57^ such an automated CT signature may be used in the future to personalize high-precision radiation therapy of prostate cancer, e.g. by means of early response assessment^58–60^. Beyond adaptive workflows, radiomic features extracted from AI-based segmentations may enable longitudinal quantitative image analysis also in non-adaptive radiotherapy settings using high-quality hCBCT-imaging.

This study has several limitations that must be taken into account. Since the study was performed with a monocentric population of 50 patients, further investigation is required for validation. Furthermore, different cancer stages as potential confounding factors need to be taken into account, potentially influencing quantitative imaging parameters, especially of the structures of the target volume, such as prostate, and seminal vesicles. Nevertheless, the analysis includes a homogeneous cohort of 100 CT/hCBCT scans for high-precision radiotherapy (RT) at the eLinac. The time comparison is based on time per slice. However, the AI autocontouring scans the entire field of view (FOV), while manual contouring or contouring correction directly jumps to relevant slices. Therefore, the slice-based analysis may be advantageous for the manual contouring assessment. Further, the results presented here are specific to the applied hCBCT preset “Pelvis” with iCBCT Acuros reconstruction. Further exploration of different presets combined with different reconstruction modes and their respective topographic scan regions or tumor sites is required.

Regarding the contouring performed, analyses including a larger number of physicians would be required to further investigate the impact of experience level and interobserver variability. While the inclusion of two observers reflects a realistic clinical scenario, it limits the generalizability of the findings. The interobserver analyses were exploratory and, due to the limited overlap cohort size per modality, were statistically powered primarily to detect large and consistent interobserver effects. Given the substantial variability of paired time differences, moderate or heterogeneous effects may not have reached statistical significance. Accordingly, these findings should be interpreted with caution and confirmed in larger multi-reader cohorts. In addition, intraobserver variability should be considered a potential source of bias. This aspect has been partially addressed in a previous study by Cvachovec and Bicu et al. for prostate segmentation and should be re-evaluated for other anatomical structures and AI-supported segmentations in future work^19^. Importantly, overlap-based metrics such as DSC are inherently volume dependent and may overestimate apparent instability in small structures; therefore, HD95 was incorporated to provide a complementary, volume-independent assessment of segmentation accuracy.

A further limitation is that all segmentations were performed in a controlled study setting rather than during routine online adaptive radiotherapy. As such, the delineations represent a simulation of the clinical workflow and may not fully capture time pressure, daily anatomical variability, longitudinally changing tissue demarcation as demonstrated in a previous analysis, or operational constraints inherent to real-world aRT^11^. Consequently, DSC and COM deviations obtained in this study may differ from values achievable in daily clinical practice. Furthermore, contouring was based exclusively on CT or hCBCT imaging. Therefore, future studies should integrate the feasibility of co-registered MR imaging for defining soft tissue organs, e.g., the prostate, as in the routine radiation planning process, into the analysis. While the stability of radiomic features was assessed for selected VOIs, bias from the different filling statuses of hollow organs may impact the analysis. While wall-focused analyses are clinically relevant, they are time-intensive and the wall itself is deformation-dependent, thus still partly driven by filling. Future work should standardize preparation (e.g., bladder/rectum protocols), record filling metrics, and model them as covariates. Additionally, future studies should evaluate different software solutions, as the analysis presented here used only one software tool for radiomic feature extraction. Consequently, a broader spectrum of radiomic features with different image preprocessing steps should be analyzed. This study lacks the analysis of a potential link between radiomics stability and geometric accuracy. Future work should explicitly test whether more stable features are associated with better geometric agreement (and, secondarily, more accurate on-couch dose) to assess inferential conclusions about radiomics–segmentation dependencies.

## Conclusion

In summary, this study is the first to comprehensively compare AI-based autocontouring during adaptive radiotherapy workflow and their derived radiomic features of the novel hCBCT-based imaging compared to diagnostic planning CT. The study could demonstrate distinct patterns for robust AI-only derived segmentations and radiomic features that may serve for personalized treatment adjustments and may be applied during adaptive radiotherapy. These trends may pave the way for a new standard of automation in targeted, adaptive treatment planning for high-precision radiotherapy approaches and even contribute to the integration of stable radiomic biomarkers into adaptive radiotherapy workflows.

## Data Availability

The data used and generated in this study may be made available, subject to ethical and data protection considerations, upon reasonable request on an individual basis. Please contact Constantin Dreher, MD (E-mail: constantin.dreher@umm.de) to request the data.
